# Automated Detection of Cancer-Suspicious Findings in Japanese Radiology Reports with Natural Language Processing: A Multicenter Study

**DOI:** 10.1007/s10278-024-01338-w

**Published:** 2025-01-22

**Authors:** Kento Sugimoto, Shoya Wada, Shozo Konishi, Junya Sato, Katsuki Okada, Shoji Kido, Noriyuki Tomiyama, Yasushi Matsumura, Toshihiro Takeda

**Affiliations:** 1 Department of Medical Informatics, Osaka University Graduate School of Medicine, 2-2 Yamadaoka, Suita, 565-0871 Osaka, Japan; 2 Department of Transformative System for Medical Information, Osaka University Graduate School of Medicine, 2-2, Yamadaoka, Suita, 565-0871 Osaka, Japan; 3 Department of Artificial Intelligence in Diagnostic Radiology, Osaka University Graduate School of Medicine, 2-2, Yamadaoka, Suita, 565-0871 Osaka, Japan; 4 Osaka University Institute for Radiation Sciences, 2-2, Yamadaoka, Suita, 565-0871 Osaka, Japan; 5 Osaka University Graduate School of Medicine, 2-2, Yamadaoka, Suita, 565-0871 Osaka, Japan; 6 Department of Radiology, Osaka University Graduate School of Medicine, 2-2, Yamadaoka, Suita, 565-0871 Osaka, Japan; 7 National Hospital Organization Osaka National Hospital, 2-1-14 Hoenzaka Chuo-ku, 540-0006 Osaka, Japan

**Keywords:** Natural language processing, Information extraction, Radiology report, Actionable findings

## Abstract

Missed critical imaging findings, particularly those indicating cancer, are a common issue that can result in delays in patient follow-up and treatment. To address this, we developed a rule-based natural language processing (NLP) algorithm to detect cancer-suspicious findings from Japanese radiology reports. The dataset used consisted of chest and abdomen CT reports from six institutions. Reports from our institution were used for algorithm development and internal evaluation, while reports from the other five institutions were used for external evaluation. To create the gold standard, reports were annotated by two experienced physicians. Data were statistically analyzed using precision, recall and F1 score with 1000 bootstrap iterations. BERT was used as a baseline deep learning model, and its performance was compared with the proposed rule-based method. At the report level of detection, the overall precision, recall, and F-1 score were 0.886, 0.886, and 0.883, respectively, for the rule-based algorithm, which were higher than those of the deep learning algorithm (0.851, 0.679, and 0.733). The overall results include both internal and external validation data. For the internal validation set, the precision, recall, and F-1 score were 0.929, 0.929, and 0.927, respectively. For the external validation set, the precision, recall, and F-1 score were 0.875, 0.879, and 0.873, demonstrating generalizability. In conclusion, we show the rule-based NLP algorithm exhibited a high performance in detecting cancer-suspicious findings from multi-institutional CT reports.

**Supplementary Information:**

The online version contains supplementary material available at 10.1007/s10278-024-01338-w.

## Introduction

Radiology reports are important documents for radiologists to communicate with referring physicians. Although radiologists document examination results in their report, busy referring physicians may overlook the content [1]. These communication problems were among the serious medical errors in the past, resulting in delays or missed patient follow-up [2, 3]. Failure to properly follow-up and treat serious diseases such as cancer has led to malpractice lawsuits [4].

To address this issue, the American College of Radiology (ACR) formed a work group and issued a statement [5]. They referred to findings that require special communication with the referring physician as “actionable findings.” They also stated that direct verbal communication (e.g., telephone) is appropriate for urgent critical findings, such as intracranial hemorrhage or arterial dissection. Conversely, they suggested that information technology may be effective for non-urgent critical findings, such as cancers, because such findings require reliable notification to referring physicians in a timely manner.

Although existing notification systems are potentially effective, they increase the workload of radiologists and rely on their subjective judgment in identifying actionable findings [1, 6, 7]. To automate this process, Natural Language Processing (NLP) offers a promising solution [8–14]. This solution has two main approaches: rule and machine learning based. The rule-based approach can be implemented using several methods, such as those based on specific phrases or terms (e.g., “follow-up”) [8–10] and those that rely on the syntactic rules of sentences [11]. It is a simple approach that provides high interpretability. However, it heavily relies on the radiologist’s writing style; consequently, detecting findings without specific phrases or terms becomes challenging. By comparison, a machine learning-based approach can detect actionable findings based on the context and textual features [12–15]. While advanced deep learning models exhibit a promising performance, they require extensive manual dataset preparation and often lack interpretability. A comparison of both approaches is shown in Table 1.

**Table 1:** Comparison of rule-based and machine learning-based approaches

	Rule-based detection	Machine learning-based detection
Description	Detects actionable findings based on phrases or terms	Detects actionable findings based on the textual features
Strengths	✓ Work without large dataset ✓ High interpretability	✓ Capture information based on context ✓ Easy to scale
Weaknesses	✓ Limited to phrases or terms ✓ Difficult to scale	✓ Needs large labeled dataset ✓ Difficult to interpret

Here, we propose a new rule-based NLP algorithm to cover a wide range of actionable findings by incorporating context-based semantic information as well as specific phrases and terms. Through this approach, the high interpretability of rule-based systems can be maintained, and a more flexible detection can be achieved by considering the meaning of text within its context. In this study, we focused on findings suggestive of cancer rather than any actionable findings. We categorized them into three levels based on the degree of priority (high actionable, low actionable, and no actionable). Furthermore, to identify cancer-suspicious location within specific parts of the body, we categorized the findings according to anatomical location (e.g., lung, breast, pancreas, etc.). We used multi-institutional radiology reports to validate the performance and generalizability of the algorithm.

## Materials and Methods

This study was approved by our institutional review board (Permission number: 24017). Informed consent was waived because of the retrospective nature of the study.

### Rule-Based Algorithm

#### System Pipeline

**Fig. 1:**
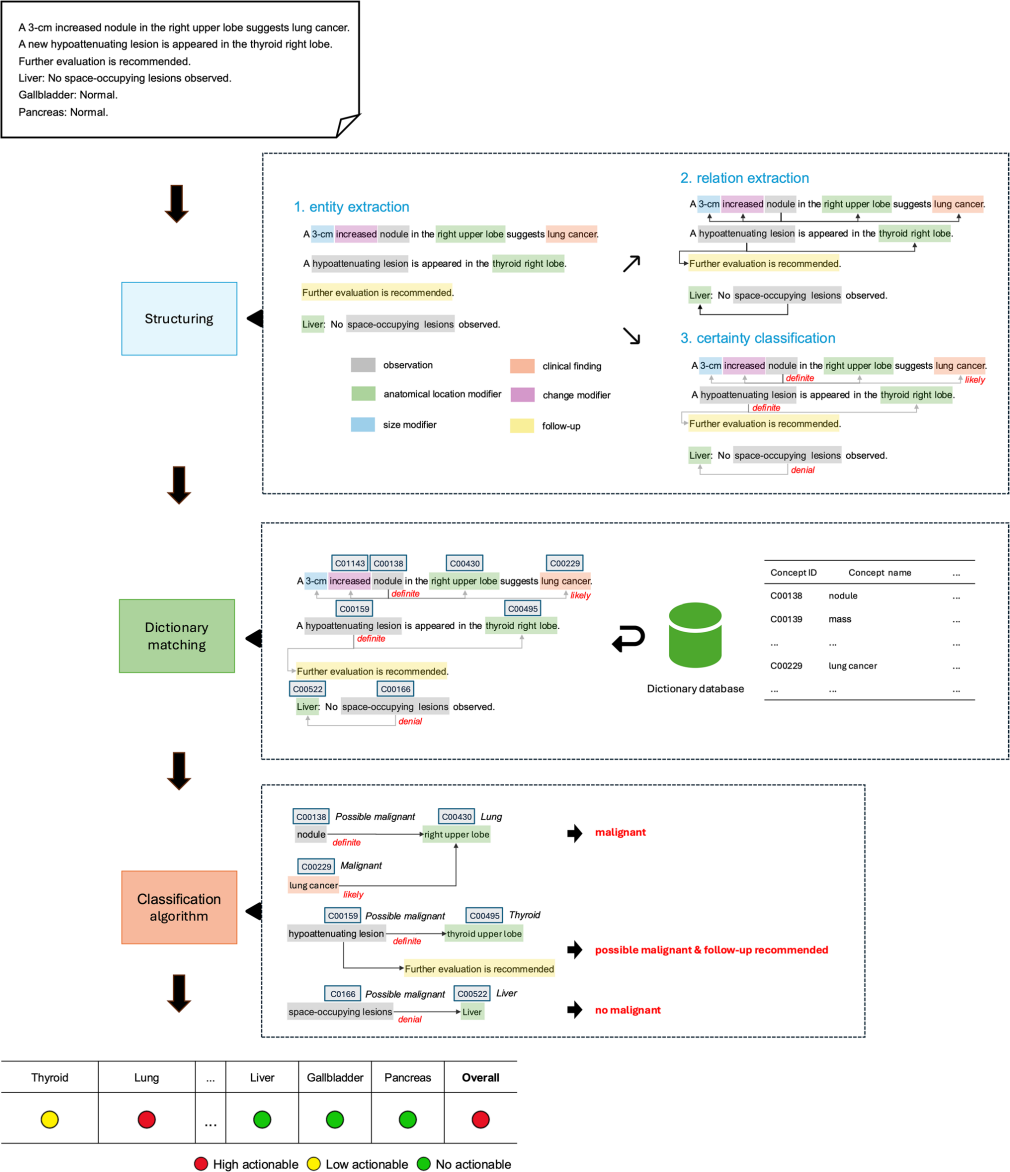
Overview of our natural language processing (NLP) system pipeline. The system is divided into several modules: 1) Entity extraction, 2) Relation extraction, 3) Certainty classification, Dictionary matching, and the Classification algorithm. First, free-text reports are structured and processed through entity extraction, where observations, clinical findings, and their modifiers are identified. Then, relation extraction and certainty classification are applied to determine the relationship between entities and assign diagnostic certainty labels (e.g., “definite,” “likely,” “denial”). Next, the structured text is matched against the dictionary database to assign concept codes. Finally, the classification algorithm groups findings into categories such as “malignant,” “possible malignant & follow-up recommended,” and “no malignant.” The final results for each anatomical location are visualized as actionable labels (high, low, or no actionable) at the bottom of the figure

Our overall system pipeline is illustrated in Fig. 1. First, our system converted a free-text report into a predefined structured schema. Second, clinical terms in a structured report were matched with our developed dictionary to assign concept codes. Finally, cancer-suspicious findings were detected by classification algorithm.

#### Structuring

Radiology reports usually have a free-text format; as such, extracting clinical information becomes challenging. To represent reports into a fixed-structured format, Sugimoto et al. [16] proposed an information model with three clinical entities: observation, clinical finding, and modifier entities. They developed an NLP system comprising entity extraction, relation extraction, and certainty classification modules. The brief description of each module in their system is as follows:

- In entity extraction, clinical terms such as observations (e.g., nodule), clinical findings (e.g., lung cancer), and their modifiers (e.g., upper lobe, 3-cm) are extracted from free-text radiology reports [16].
- In relation extraction, correct relations between the extracted entities are obtained [17].
- In certainty classification, a five-level diagnostic certainty scale (definite, likely, may represent, unlikely, denial) is assigned to the observation and clinical finding entities [18].

**Fig. 2:**
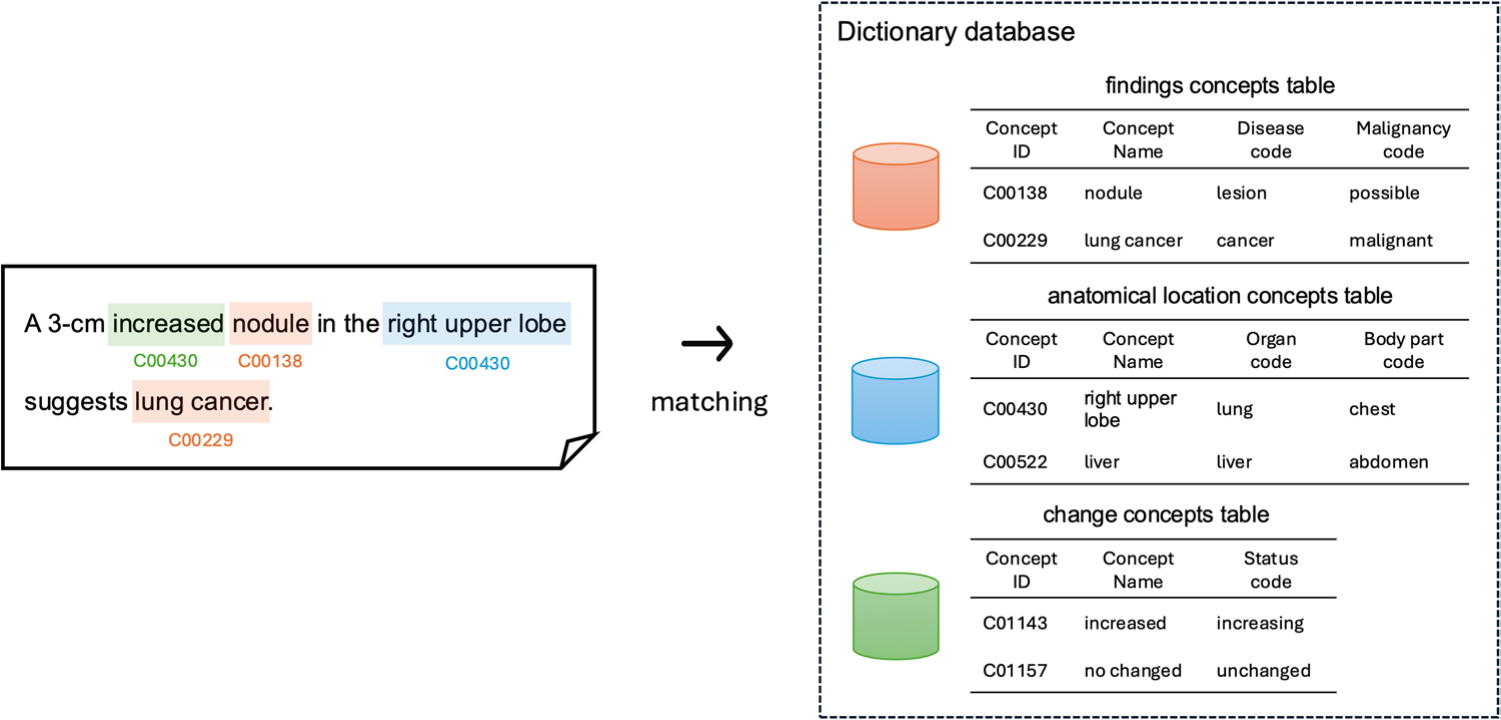
Overview of the dictionary matching process. The example sentence, “A 3-cm increased nodule in the right upper lobe suggests lung cancer,” is processed by matching entities to the dictionary database. Each word or phrase is matched to the corresponding concepts in three tables: findings concepts table, anatomical location concepts table, and change concepts table. For instance, “nodule” is matched to a concept in the findings concepts table (C00138), “right upper lobe” is matched to a concept in the anatomical location concepts table (C00430), and “increased” is matched to a concept in the change concepts table (C01143)

They described their methods in detail in their research [16–18]. We extended this model to include follow-up entities because of their importance in identifying actionable findings [8, 13]. Figure 2 shows our customized information model.

### Dictionary Matching

Because of insufficient resources of Japanese clinical terms and phrases [19], we constructed a Japanese radiological dictionary database for this study. Our database consists of two main types of tables: surfaces and concepts. The surface table contains phrases and words that appear in reports, while the concept table standardizes these phrases and words in the surface table. The concept table is divided into the following three categories:

- In the finding concept table, extracted words or phrases as either observation or clinical finding entities are matched. This table includes the attributes of a disease code based on ICD-10 and a malignancy code that indicates the likelihood of malignancy (possible, benign, indeterminate, malignant).
- In the anatomical location concept table, extracted words or phrases as anatomical location modifier entities are matched. This table includes the attributes of an organ code and a body part code.
- In the change concept table, words or phrases extracted as change modifier entities are matched. This table includes a standardized status code attribute (increasing, decreasing, new, unchanged).

Words and phrases extracted as specific entities (observation, clinical finding, anatomical location modifier, change modifier) in a report were string-matched with the dictionary to look up their concept id. The process of dictionary matching is visualized in Fig. 2. The details of our dictionary construction process are provided in supplementary materials.

### Classification Algorithm

Before a classification algorithm was described, cancer-suspicious findings were clearly defined. In this study, they are defined as explicit clinical findings indicating malignancy and any radiological observations that suggest the potential occurrence of cancer. Here, these findings include not only specific phrases or terms but also the specific context of a report. Our previous institutional reports were analyzed to reference relevant radiology reports. Through our radiology reporting system, radiologists can flag reports that include critical findings during routine clinical practice. Then, cancer-suspicious reports were manually collected from the flagged reports. Our analysis revealed four distinct writing patterns flagged by radiologists as cancer-suspicious findings. The four patterns include:

- **P1 (malignant)** are findings explicitly indicating a suspicion of malignancy;
- **P2 (indeterminate)** are findings where the malignancy cannot be definitively determined
- **P3 (follow-up recommended)** are findings not explicitly suggesting malignancy but prompting further investigation or follow-up due to clinical suspicion; and
- **P4 (increasing or new lesion)** are findings not explicitly suggesting malignancy but demonstrating growth or new appearance, raising concern for malignancy.

**Fig. 3:**
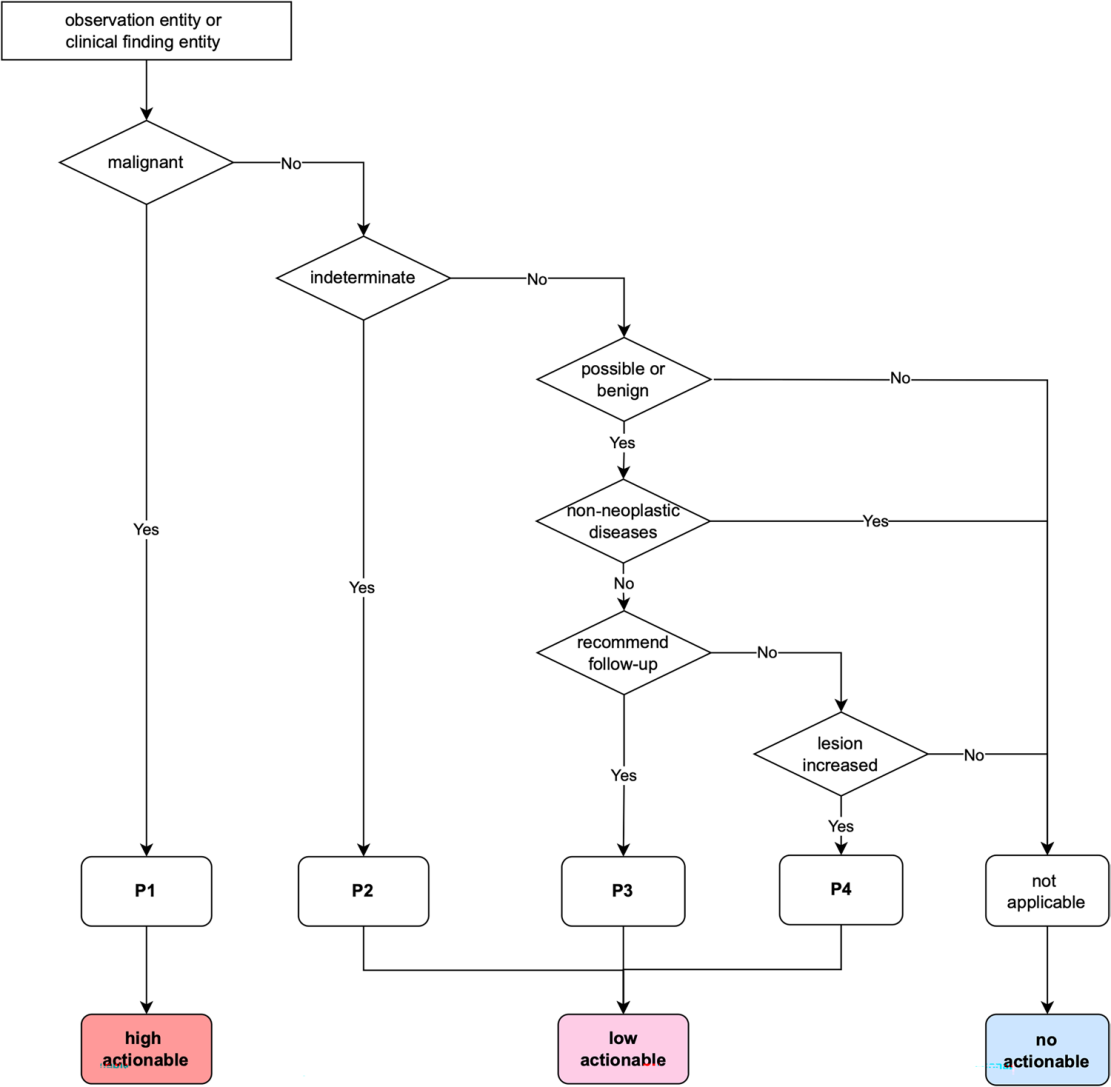
Flowchart of classification algorithm. The algorithm classifies findings suggestive of cancer into four patterns: P1, P2, P3, and P4

A classification algorithm was implemented to identify these four patterns from reports. Our algorithm detects cancer-suspicious terms based on the observation and clinical finding entities and their modifier entities identified during the structuring step. Figure 3 provides a visual representation of this step, outlining the flow of the classification algorithm.

First, observation and clinical finding entities that explicitly negated the presence of findings (e.g., “no evidence of malignancy”) were excluded by using the diagnostic certainty scale of each entity. This scale, provided by the certainty classification module in the structuring step, categorizes the findings into a five-level scale: definite, likely, may represent, unlikely, and denial. We specifically excluded the findings labeled as “denial.”

Second, for the remaining observation and clinical entities, classification rule was developed to categorize them into one of the four patterns or as not applicable. Specifically, entities were classified according to their malignancy code that was mapped to finding concepts during the previous dictionary matching step: “malignant” is classified as **P1**, and “indeterminate” is classified as **P2**. While P1 and P2 are determined explicitly based on terms such as “cancer” or “tumor,” P3 and P4 are determined based on terms and their surrounding context (e.g., “the nodule requires follow-up”). Therefore, P3 and P4 were classified according to observation and clinical entities and their modifiers. Specifically, entities that were mapped to finding concepts with a malignancy code of “benign” or “possible,” and had follow-up entity modifiers were classified as **P3**. Entities with change entity modifiers and their status code of “new” or “increasing” were classified as **P4**. Entities related to non-neoplastic disease codes (e.g., pneumonia) were excluded although they met the **P3** or **P4** criteria. Entities that were not mapped to a finding concept or were mapped a findings concept with an empty malignancy code were classified as “not applicable.” The classification rules and examples for each pattern are presented in Table 2.

**Table 2:** Classification rules and examples for each pattern (The bold with underline text highlights the key elements in each example for classification rules)

Pattern	Rule	Category	Example
P1	1. The malignancy code of the finding is “malignant”	high actionable	suspected **lung cancer**_**[malignant]**_ (original) **肺癌**_**[malignant]**_を疑う
P2	1. The malignancy code of the finding is “indeterminate”	low actionable	suspected **ovarian tumor**_**[indeterminate]**_ (original) **卵巣腫瘍**_**[malignant]**_を疑う
P3	1. The malignancy code of the finding is “possible” or “benign” 2. A finding is related to a “follow-up entity”	low actionable	A **hypoattenuating lesion**_**[possible]**_ is seen in the thyroid right lobe. **Further evaluation is recommended**. (original) 甲状腺に**低吸収域**_**[possible]**_を認める。**追加検査をご検討ください**。
P4	1. The malignancy code of findings is “possible” or “benign” 2. A finding is related to “change modifier entity,” and its status code is “new” or “increasing”	low actionable	A **lung nodule**_**[possible]**_ has **increased**_**[increasing]**_ in size. (original) **肺結節**_**[possible]**_が**増大**_**[increasing]**_しています。

Lastly, P1 findings were labeled “high actionable,” P2, P3, and P4 findings were “low actionable,” and those not matching any pattern were “no actionable.” The preprocessing and the classification algorithm were applied to the findings and impression sections of the reports.

The anatomical location of a given finding was identified by referencing either the finding itself or any related anatomical location modifier entities.

### Deep Learning-Based Algorithm

As a baseline, we developed a deep learning-based classification algorithm. We applied BERT [20], a widely used NLP model. As a straightforward method, we formulated this task as a multi-class classification problem by using a free-text report as input and a predicted actionable label as output. We used UTH-BERT [21], a publicly available pre-trained Japanese clinical BERT model, and fine-tuned it with labeled reports. We provide more details on the setting of the deep learning-based method in supplementary materials.

### Dataset

Multi-institutional chest and abdomen CT reports from January 2020 to December 2023 were used. The dataset was divided into development (January 2020–June 2023), and test sets (July 2023–December 2023). The test set was composed of internal and external validation sets from our institution and five other institutions, respectively.

### Development Sets

A total of 955 radiology reports from our institution were used for algorithm development and refinement. For the deep learning-based algorithm, these reports were used to fine-tune the model.

### Test Set

A total of 900 reports were collected from the Japan Medical Imaging Database (J-MID), with 150 reports from each of the following institutions: Kyoto University Hospital, Okayama University Hospital, Osaka University Hospital, Juntendo University Hospital, Hokkaido University Hospital, and Ehime University Hospital. The internal validation set was composed of radiology reports from our institution, where the algorithm was initially developed. For external validation, reports from five other institutions, which were not involved in the algorithm development, were used to assess its generalizability.

### Annotations

Reports were annotated by two experienced physicians who read the findings and impression sections of the reports. After a guideline for cancer-suspicious findings was provided, annotators were asked to read the findings and impression sections of the reports and label them as “high actionable,” “low actionable,” or “no actionable.” They were instructed to assign actionable labels for each anatomical location (e.g., lung, breast, pancreas, etc.), which were predefined by experts. Cohen’s kappa [22] was used to measure the inter-annotator agreement (IAA) score. Initially, the experts had many disagreements because of differences in their knowledge and experience. To improve the agreement score, sample reports were repeatedly provided, and the guidelines were refined. Consequently, the annotators’ understanding of the task was enhanced, achieving the IAA scores of 0.913 at the report level and 0.855 at the anatomical location level in the test set, indicating a very high agreement [23]. Disagreements between the annotators were resolved by discussion.

### Statistical Analysis

Precision, recall, and F1 score were used to evaluate our algorithm. Overall results were calculated using a macro average, and 95% confidence intervals (CI) were calculated by 1000 bootstrap iterations.

## Results

Our algorithm and the evaluation dataset were labeled at the anatomical location level. Therefore, in the evaluation at the report level, the following rules were used to assign labels to the reports:

- The report was labeled as “high actionable” if at least one anatomical location was labeled with “high actionable.”
- The report was labeled as “low actionable” if no regions were labeled with “high actionable,” but at least one region was labeled with “low actionable.”
- The report was labeled as “no actionable” if no regions were labeled with either “high actionable” or “low actionable.”

Table 3 shows the statistical comparison results between the rule-based and deep learning-based algorithms at the report level. The rule-based algorithm achieved a precision, recall, and F-1 score of 0.886 (95% CI, 0.850–0.918), 0.886 (95% CI, 0.852–0.920), and 0.883 (95% CI, 0.850–0.914), respectively; conversely, the deep learning-based algorithm achieved 0.851 (95% CI, 0.801–0.896), 0.679 (95% CI, 0.635–0.720), and 0.773 (95% CI, 0.688–0.776), respectively. The difference (delta) between the rule-based and deep learning-based algorithms in Table 3 indicates that performance of the rule-based was statistically higher than that of the deep learning-based algorithm. For the remainder of the experiments, we evaluated the rule-based algorithm to assess its detailed performance.

**Table 3:** Comparison results between rule-based and deep learning-based algorithm

Algorithm	Precision	Recall	F1-score
Rule-based	0.886 (0.850, 0.918)	0.886 (0.852, 0.920)	0.883 (0.850, 0.914)
Deep learning-based	0.851 (0.801, 0.896)	0.679 (0.635, 0.720)	0.733 (0.688, 0.776)
Delta	0.035 (−0.025, 0.094)	0.208 (0.157, 0.261)	0.151 (0.099, 0.205)

Data in parentheses are 95% confidence intervals

The evaluation results at the report level using the entire test set from the six institutions are presented in Table 4. The overall precision, recall, and F-1 score were 0.886 (95% CI, 0.850–0.918), 0.886 (95% CI, 0.852–0.920), and 0.883 (95% CI, 0.850–0.914), respectively. The overall precision, recall, and F-1 score for “high actionable” were 0.862 (95% CI, 0.818–0.905), 0.986 (95% CI, 0.968–1.000), and 0.920 (95% CI, 0.892–0.946), respectively; for “low actionable,” they were 0.813 (95% CI, 0.714–0.902), 0.724 (95% CI, 0.627–0.816), and 0.764 (95% CI, 0.685–0.835), respectively. For “no actionable,” the overall precision, recall, and F-1 score were 0.983 (95% CI, 0.972–0.993), 0.950 (95% CI, 0.932–0.966), and 0.966 (95% CI, 0.955–0.976), respectively. “High actionable” and “no actionable” demonstrated a high overall performance with small statistical variations.

**Table 4:** Evaluation results at the report level across six institutions

Label	Precision	Recall	F1-score	N
High actionable	0.862 (0.818, 0.905)	0.986 (0.968, 1.000)	0.920 (0.892, 0.946)	210
Low actionable	0.813 (0.714, 0.902)	0.724 (0.627, 0.816)	0.764 (0.685, 0.835)	82
No actionable	0.983 (0.972, 0.993)	0.950 (0.932, 0.966)	0.966 (0.955, 0.976)	608
Total	0.886 (0.850, 0.918)	0.886 (0.852, 0.920)	0.883 (0.850, 0.914)	900

Data in parentheses are 95% confidence intervals

**Fig. 4:**
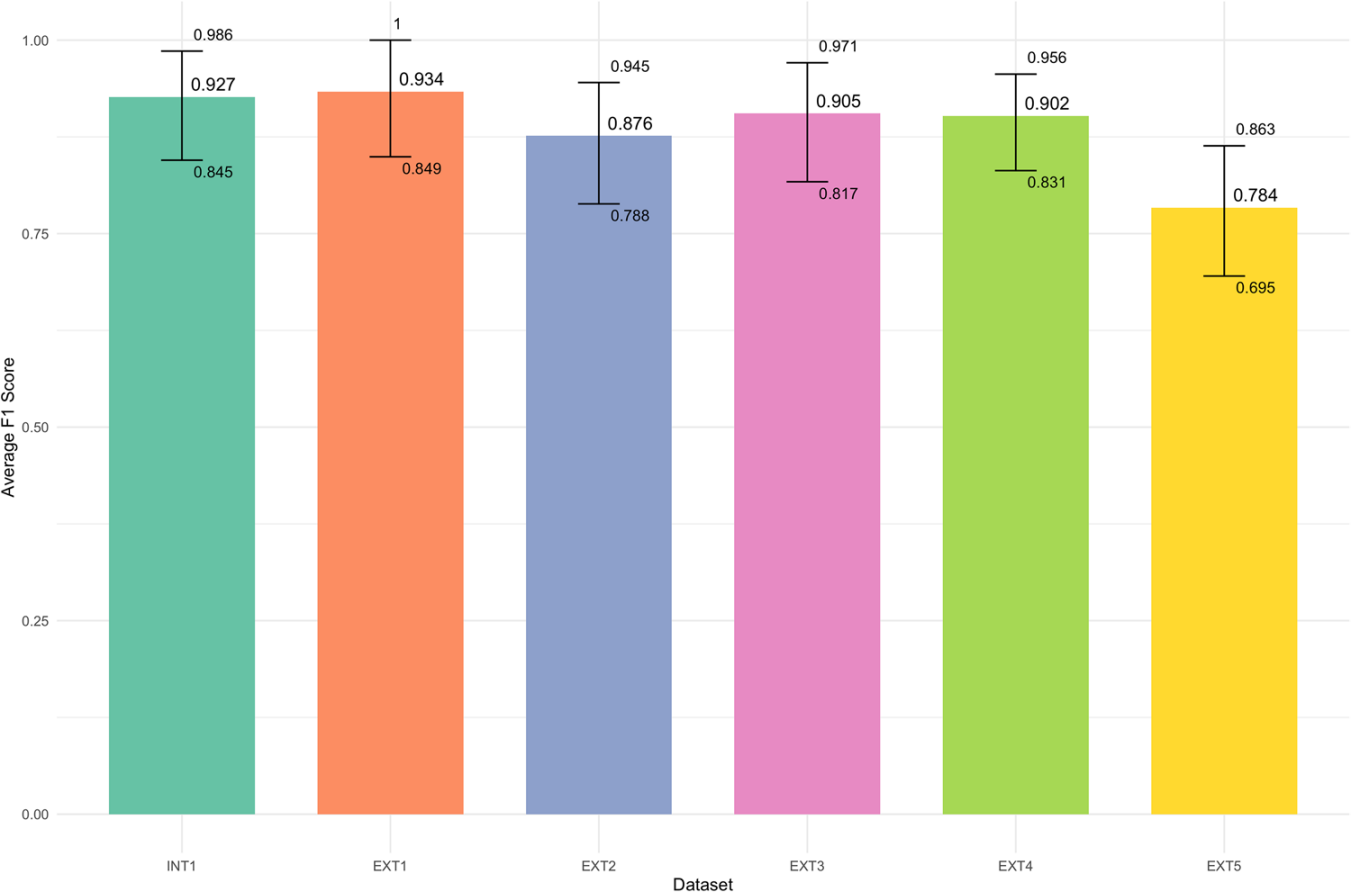
Statistical comparison of performance by the institutions. “INT” represents the internal dataset, and “EXT” represents the external dataset

Table 5 compares the results between the internal set from our institution and the external set from the other five institutions. In the internal validation set, the precision, recall, and F-1 score were 0.929 (95% CI, 0.840–0.997), 0.929 (95% CI, 0.841–0.997), and 0.927 (95% CI, 0.845–0.986), respectively. In the external validation set, the precision, recall, and F-1 score were 0.875 (95% CI, 0.837–0.909), 0.879 (95% CI, 0.842–0.916), and 0.873 (95% CI, 0.837–0.907), respectively. For all metrics, the internal set showed approximately 0.05 higher values; however, the difference in confidence intervals (delta) indicated no statistical significance. Figure 4 presents the comparison result of F-1 scores by the institutions. “INT” and “EXT” refer to the internal and external sets, respectively. The external dataset, except for EXT5, demonstrated high performance.

**Table 5:** Comparison results between the internal and external sets

Dataset	Precision	Recall	F1-score	N
Internal	0.929 (0.840, 0.997)	0.929 (0.841, 0.997)	0.927 (0.845, 0.986)	150
External	0.875 (0.837, 0.909)	0.879 (0.842, 0.916)	0.873 (0.837, 0.907)	750
Delta	0.053 (−0.049, 0.135)	0.050 (−0.048, 0.132)	0.053 (−0.034, 0.126)	900

Data in parentheses are 95% confidence intervals

The experimental results by anatomical location are shown in Table 6. The overall precision, recall, and F1 score were 0.821 (95% CI, 0.785–0.855), 0.843 (95% CI, 0.812–0.876), and 0.816 (95% CI, 0.785–0.845), respectively. The performance at the anatomical location level was lower than that at the report level because this evaluation required strict agreement at the anatomical location level. Details of the results by anatomical location are shown in Supplemental Table 2.

**Table 6:** Evaluation results at the anatomical location level across six institutions

Label	Precision	Recall	F1-score
High actionable	0.816 (0.777, 0.857)	0.841 (0.798, 0.882)	0.803 (0.763, 0.840)
Low actionable	0.649 (0.553, 0.730)	0.695 (0.605, 0.786)	0.650 (0.568, 0.725)
No actionable	0.998 (0.997, 0.998)	0.994 (0.993, 0.995)	0.996 (0.995, 0.997)
Total	0.821 (0.785, 0.855)	0.843 (0.812, 0.876)	0.816 (0.785, 0.845)

Data in parentheses are 95% confidence intervals

## Discussion

### Error Analysis

Table 3 shows that the performance of the rule-based algorithm was significantly better than that of the deep learning algorithm. Classifying cancer-suspicious findings is a complex task that involves recognizing cancer-related terms and detecting negations. We considered that the performance of the deep learning algorithm was lower likely because of the insufficient sample size of the training set for solving such a complex task. For example, Yang et al. [24] recommended that at least 1000 samples are required for the classification of radiology reports by using the BERT model. According to their research, our training set, which consisted of 955 samples, was likely insufficient to obtain satisfactory performance. Although increasing the sample size could potentially improve performance, it would involve additional costs for dataset preparation, and further consideration of this issue was beyond the scope of this study.

The macro-average F1 score for “low actionable” was 0.764, which was lower than that for “high actionable” and “no actionable” (Table 4). “High actionable” is a straightforward task that primarily identifies phrases such as “cancer,” while “low actionable” is more challenging because it requires understanding the context of the findings. We observed that annotation disagreements were most frequent in “low actionable,” showing that even human annotators, including experienced physicians, struggled to determine “low actionable.” This observation also highlighted that even experienced physicians have trouble determining appropriate actions for relatively less important findings that still require follow-up. For example, in the follow-up of a lesion suspected of a non-neoplastic disease, one physician determined it as “no actionable,” while another identified it as “low actionable” (Table 7, no. 1) because the possibility of malignancy could not be completely ruled out. Although the characteristics of potentially malignant lesions had numerous patterns, we failed to cover them sufficiently because of the limited size of the training and development sets. Imaging features related to contrast enhancement (e.g., non-enhancing area) may indicate the possibility of malignancy, but our dictionary did not assign malignancy codes to some of those phrases. Furthermore, some radiologists may include certain diagnoses only in the impression section (Table 7, no. 2, 3). Although annotators could integrate information from the findings and diagnostic sections, our algorithm could not perform this integration because it independently extracted findings from each section.

**Table 7:** Error analysis examples

No	Findings	Impression	Gold	Prediction
1	Multiple nodules in the lower lobe of the left lung probably suggest pulmonary cryptococcosis. Additional follow-up is recommended. (original) 左肺下葉の多発結節は肺クリプトコッカス症を疑います。追加のフォローください。	Multiple nodules. (original) 多発結節	Low actionable	No actionable
2	Multiple small nodules in both lungs are increasing in size. (original) 両肺に見られる多発小結節は増大しています。	Suspected nontuberculous mycobacterial lung disease. (original) 非結核性抗酸菌症を疑います。	No actionable	Low actionable
3	Ground-glass nodules in both lungs are worsening. (original) 両肺のすりガラス結節は悪化しています。	Suspected interstitial pneumonia. (original) 間質性肺炎を疑います。	No actionable	Low actionable
4	A nodule in the lower lobe of the left lung is suggestive of post-inflammatory change, but differentiation from metastasis is difficult. (original) 左肺下葉の結節は炎症後変化を疑います。しかし、転移の鑑別は困難です。	A nodule in the left lung: suggestive of post-inflammatory change. (original) 左肺結節:炎症後変化を疑います。	Low actionable	High actionable
5	Multiple uterine fibroids are suspected. Exclusion of malignancy is necessary. (original) 多発子宮筋腫が疑われます。悪性の除外が必要です。	Suspicious of multiple uterine fibroids. (original) 多発子宮筋腫の疑い。	Low actionable	High actionable

We demonstrated that the internal and external sets did not significantly differ based on the difference in their confidence intervals (Table 5); the F-1 scores did not also significantly vary between the internal set and four out of the five external sets (Figs. 4, 5). Error analysis revealed that the differences were attributed to variations in writing styles among the institutions. Many false positives in the external validation set contained problem lists (e.g., “# squamous cell carcinoma”) in the report headers. The annotators labeled it as “no evidence” because it was not found in the image examination. We also observed several false positive cases where malignant phrases were written, but the annotators assigned them as “low actionable” because the whole context did not explicitly indicate any cancer suspicion (Table 7, no. 4, 5). This error pattern was particularly frequent in EXT5, which explained the lower performance of EXT5.

The macro-average F1 score of the classification performance at the anatomical location level was approximately 6.7% lower than that at the report level in the entire dataset (Tables 3 and 4). The most frequent error is a case wherein the lesion’s location is indicated with positional words, as in the following example.

- The soft tissue opacity is increased **near the right common iliac artery**.
- Lymph node enlargement **around the left gastric artery** suggests metastasis.

On the basis of medical knowledge, annotators labeled these parts as “peritoneum” and “abdominal lymph nodes,” respectively. In the first example, the algorithm predicted “other” because “near the right common iliac artery” was not found in our anatomical location dictionary. In the second example, the algorithm predicted “kidney” when our anatomical location dictionary was searched. The lesion’s location is often indicated by positional words, especially in abdominal CT reports. Therefore, we considered expanding the dictionary to accommodate these cases.

### Limitations and Future Work

Our study has several limitations. First, because of the burden on annotators and time constraints, we only evaluated 150 samples per institution. A larger sample size may be required to assess a wider variety of report styles. Additionally, bootstrap confidence intervals should be measured with a larger number of samples to enable more accurate comparisons. Second, only chest and abdominal CT reports were the modalities and body parts used in the dataset. We are currently evaluating the applicability of our algorithm to chest radiograph reports. We believe that It is feasible because findings are often simpler in radiograph reports than in chest CT reports.

Lastly, although our algorithm achieved a high performance, a gap remains between our algorithm and a practical system for clinical use. In this study, we conducted experiments to classify cancer-suspicious findings from a single report. Therefore, we did not consider the chronological progresses in the patient’s clinical condition. Notifying the users would be more effective only when a cancer-suspicious finding was detected for the first time in each anatomical location rather than notifying them for every report. A beneficial clinical decision support system should adjust notification frequencies to reduce alert fatigue and alert overrides [25–27].

In conclusion, we developed a rule-based NLP system to detect cancer-suspicious findings from free-text radiology reports. We created practical decision rules and implemented an algorithm based on the analysis of past actionable reports. We experimentally demonstrated that the algorithm is sufficiently feasible and generalizable. Future studies should evaluate the feasibility of our system in real-world clinical practice.

## Supplementary Information

ESM 1 (DOCX 37.2 KB)
