# Supplementary material for: Automated Detection of Cancer-Suspicious Findings in Japanese Radiology Reports with Natural Language Processing: A Multicenter Study
Source: J Imaging Inform Med. 2025 Jan 22;38(5):3375–85. doi: 10.1007/s10278-024-01338-w (PMC12572415; doi:10.1007/s10278-024-01338-w)
Supplement: Supplementary file 1 — (DOCX 37.2 KB) [file 10278_2024_1338_MOESM1_ESM.docx]

# **Supplemental Information**

**Dictionary construction**

Initially, we defined a dictionary database that included the following: finding concepts containing clinical concepts related to lesions and diagnoses, anatomical location concepts containing clinical concepts related to organs and body parts, and change concepts containing clinical concepts related to progress and status. Our definitions were based on RadLex[1].

Next, the words and phrases were mapped to the defined concept dictionary. we collected a large number of words and phrases using the following semi-automated approach:

1. We applied the entity recognition model developed by Sugimoto et al.[2] to extract clinical entities from the reports. We used approximately 260,000 CT reports from 2012 to 2020 in our institution.
2. From the extracted entities, we created a word list of findings by collecting words and phrases related to lesions and diagnoses. Similarly, we prepared an anatomical location word list, compiling words and phrases related to organs and body parts, and a change word list, compiling words and phrases related to changes. Due to limited coding resources, words and phrases occurring two times or fewer in the corpus were excluded from subsequent coding work.
3. Three medical students mapped each word or phrase to the appropriate concept. Since the words and phrases automatically extracted by machine learning may contain irrelevant words and phrases, we asked the medical students to exclude them. Disagreements were resolved by majority vote. In the final stage, a sample of the results was selected and refined by two physicians.
4. The dictionary was iteratively refined throughout the training and development phases.

### **Deep learning-based approach**

As a baseline, we adopted UTH-BERT[3], a deep learning-based approach pre-trained on Japanese medical texts. The method for morphological analysis followed their original approach. The representation of the first “[CLS]” token for classification was used, which is a straightforward sequence classification tasks introduced by the original BERT. The hyperparameters were selected empirically as follows: 5 training epochs, a batch size of 16, and the AdamW optimizer with a learning rate of 5e-5. Since all reports in our dataset had sequence lengths within 512 tokens, the maximum sequence length was set to 512.

We fine-tuned the model to classify the overall actionable label from the reports. In preliminary experiments, we attempted to classify actionable labels by anatomical location level, but the performance was significantly low due to label imbalanced (most labels at the anatomical location level were “no actionable”). Therefore, we focused on classifying the overall label instead.

### **Examples of preprocessing**

The following are examples of structuring and semantic coding in preprocessing:

#### Japanese

(Original text)

右肺上葉に3cm大の増大する結節を認め、肺癌を疑います。フォローアップをお願いします。

(structuring and semantic coding)

[

{

“type”: “Observation”,

“surface_name”: “結節”,

“concept_code”: “C00138”,

“certainty_level”: “definitive”,

“modifiers”: [

{

“type”: “Anatomical_location”,

“surface_name”: “右肺上葉”,

“concept_code”: “C00430”

},

{

“type”: “Size “,

“surface_name”: “３cm大”,

“concept_code”: null

},

{

“type”: “Change “,

“surface_name”: “増大する”,

“concept_code”: “C01143”

},

{

“type”: “Followup “,

“surface_name”: “フォローアップをお願いします”,

“concept_code”: null

}

],

“causations”: [

{

“type”: “Clinical_finding”,

“surface_name”: “肺癌”,

“concept_code”: “C00229”,

“certainty_level”: “likely”,

“modifiers”: [

{

“type”: “Followup “,

“surface_name”: “フォローアップをお願いします”,

“concept_code”: null

}

]

}

]

}

]

#### English

(Original text)

A 3-cm increased nodule in the right upper lobe suggests lung cancer. Follow-up is recommended.

(structuring and semantic coding)

[

{

“type”: “Observation”,

“surface_name”: “nodule”,

“concept_code”: “C00138”,

“certainty_level”: “definitive”,

“modifiers”: [

{

“type”: “Anatomical_location”,

“surface_name”: “ right upper lobe”,

“concept_code”: “C00430”

},

{

“type”: “Size “,

“surface_name”: “3-cm”,

“concept_code”: null

},

{

“type”: “Change “,

“surface_name”: “ increased”,

“concept_code”: “C01143”

},

{

“type”: “Followup “,

“surface_name”: “Follow-up is recommended”,

“concept_code”: null

}

],

“causations”: [

{

“type”: “Clinical_finding”,

“surface_name”: “lung cancer “,

“concept_code”: “C00229”,

“certainty_level”: “likely”,

“modifiers”: [

{

“type”: “Followup “,

“surface_name”: “Follow-up is recommended”,

“concept_code”: null

}

]

}

]

}

]

### **References**

[1] Datta S, Godfrey-Stovall J, Roberts K. RadLex Normalization in Radiology Reports. AMIA Annu Symp Proc. 2020;2020:338–47,

[2] Sugimoto K, Takeda T, Oh J-H, Wada S, Konishi S, Yamahata A, Manabe S, Tomiyama N, Matsunaga T, Nakanishi K, Matsumura Y. Extracting clinical terms from radiology reports with deep learning. J Biomed Inform. 2021 Apr 1;116:103729,

[3] Kawazoe Y, Shibata D, Shinohara E, Aramaki E, Ohe K. A clinical specific BERT developed using a huge Japanese clinical text corpus. PLoS One. 2021 Nov 9;16(11):e0259763,

### **Tables**

Supplemental Table 1 Classification performance by anatomical location

|  | high actionable | | | low actionable | | | no actionable | | |
| --- | --- | --- | --- | --- | --- | --- | --- | --- | --- |
| Organ location | precision | recall | F1-score | precision | recall | F1-score | precision | recall | F1-score |
| Thyroid | 0.000 | 0.000 | 0.000 | 0.663 | 0.853 | 0.732 | 0.999 | 0.996 | 0.997 |
| Lung | 0.897 | 0.963 | 0.929 | 0.397 | 0.593 | 0.471 | 0.992 | 0.968 | 0.980 |
| Pleura | 0.748 | 0.996 | 0.844 | 0.000 | 0.000 | 0.000 | 1.000 | 0.999 | 0.999 |
| Heart | 0.615 | 0.615 | 0.615 | 0.000 | 0.000 | 0.000 | 1.000 | 1.000 | 1.000 |
| Breast | 0.818 | 1.000 | 0.895 | 0.874 | 0.500 | 0.611 | 0.999 | 0.999 | 0.999 |
| Mediastinum | 0.167 | 0.430 | 0.222 | 0.702 | 0.875 | 0.769 | 0.998 | 0.991 | 0.994 |
| Esophagus | 0.496 | 0.629 | 0.523 | 0.000 | 0.000 | 0.000 | 0.997 | 0.998 | 0.997 |
| Stomach | 0.504 | 0.826 | 0.611 | 0.629 | 0.629 | 0.629 | 0.999 | 0.994 | 0.997 |
| Liver | 0.883 | 0.785 | 0.828 | 0.791 | 0.799 | 0.776 | 0.993 | 0.997 | 0.995 |
| Gallbladder | 0.645 | 0.645 | 0.645 | 0.000 | 0.000 | 0.000 | 1.000 | 1.000 | 1.000 |
| Bile duct | 0.868 | 0.868 | 0.868 | 0.000 | 0.000 | 0.000 | 1.000 | 1.000 | 1.000 |
| Pancreas | 0.792 | 0.889 | 0.830 | 0.907 | 0.802 | 0.849 | 0.992 | 0.995 | 0.994 |
| Spleen | 0.874 | 0.874 | 0.874 | 0.000 | 0.000 | 0.000 | 1.000 | 1.000 | 1.000 |
| Kidney | 0.643 | 0.872 | 0.729 | 0.338 | 0.874 | 0.465 | 1.000 | 0.992 | 0.996 |
| Adrenal gland | 0.994 | 0.994 | 0.994 | 0.656 | 0.891 | 0.736 | 1.000 | 0.999 | 0.999 |
| Small intestine | 0.000 | 0.000 | 0.000 | 0.000 | 0.000 | 0.000 | 1.000 | 0.999 | 0.999 |
| Large intestine | 0.421 | 0.943 | 0.561 | 0.400 | 0.636 | 0.465 | 0.999 | 0.992 | 0.995 |
| Peritoneum | 0.843 | 0.846 | 0.841 | 0.324 | 0.632 | 0.408 | 0.999 | 0.997 | 0.998 |
| Uterus | 0.500 | 0.984 | 0.646 | 0.305 | 0.419 | 0.326 | 1.000 | 0.994 | 0.997 |
| Vagina | 0.852 | 0.852 | 0.852 | 0.000 | 0.000 | 0.000 | 1.000 | 1.000 | 1.000 |
| Vulva | 0.638 | 0.638 | 0.638 | 0.000 | 0.000 | 0.000 | 1.000 | 1.000 | 1.000 |
| Ovary | 0.410 | 0.312 | 0.325 | 0.640 | 0.869 | 0.715 | 0.998 | 0.998 | 0.998 |
| Prostate | 0.992 | 0.992 | 0.992 | 0.000 | 0.000 | 0.000 | 0.999 | 1.000 | 0.999 |
| Testis | 0.000 | 0.000 | 0.000 | 0.000 | 0.000 | 0.000 | 1.000 | 1.000 | 1.000 |
| Penis | 0.000 | 0.000 | 0.000 | 0.000 | 0.000 | 0.000 | 1.000 | 1.000 | 1.000 |
| Bladder | 0.804 | 0.987 | 0.874 | 0.000 | 0.000 | 0.000 | 1.000 | 0.999 | 0.999 |
| Ureter | 0.745 | 0.949 | 0.819 | 0.000 | 0.000 | 0.000 | 1.000 | 0.999 | 0.999 |
| Urethra | 0.000 | 0.000 | 0.000 | 0.000 | 0.000 | 0.000 | 1.000 | 1.000 | 1.000 |
| Cervical lymph nodes | 0.714 | 0.549 | 0.604 | 0.000 | 0.000 | 0.000 | 0.995 | 0.998 | 0.997 |
| Thoracic lymph nodes | 0.800 | 0.601 | 0.679 | 0.000 | 0.000 | 0.000 | 0.989 | 0.997 | 0.993 |
| Abdominal lymph nodes | 0.890 | 0.441 | 0.580 | 0.000 | 0.000 | 0.000 | 0.989 | 0.999 | 0.994 |
| Pelvic lymph nodes | 0.994 | 0.557 | 0.697 | 0.000 | 0.000 | 0.000 | 0.996 | 1.000 | 0.998 |
| Bone | 0.831 | 0.880 | 0.851 | 0.000 | 0.000 | 0.000 | 0.997 | 0.997 | 0.997 |
| Others | 0.153 | 0.798 | 0.255 | 0.040 | 0.317 | 0.070 | 0.998 | 0.901 | 0.947 |
| **Macro avg** | **0.816** | **0.841** | **0.803** | **0.649** | **0.695** | **0.650** | **0.998** | **0.994** | **0.996** |
